# Longitudinal analysis of FcRL5 expression and clonal relationships among classical and atypical memory B cells following malaria

**DOI:** 10.1186/s12936-021-03970-1

**Published:** 2021-11-10

**Authors:** S. Jake Gonzales, Sebastiaan Bol, Ashley E. Braddom, Richard Sullivan, Raphael A. Reyes, Isaac Ssewanyana, Erica Eggers, Bryan Greenhouse, Evelien M. Bunnik

**Affiliations:** 1 Department of Microbiology, Immunology and Molecular Genetics, Long School of Medicine, The University of Texas Health Science Center at San Antonio, San Antonio, TX USA; 2 Department of Medicine, University of California San Francisco, San Francisco, CA USA; 3 London School of Hygiene and Tropical Medicine, London, UK; 4 Infectious Disease Research Collaboration, Kampala, Uganda; 5 Department of Neurology, UCSF Weill Institute for Neurosciences, University of California San Francisco, San Francisco, CA USA; 6 Present Address: Shape Therapeutics, 219 Terry St., Seattle, WA USA

**Keywords:** *Plasmodium falciparum*, Adaptive immune response, Humoral immunity, B cell differentiation, Infection, BCR-sequencing, B cell receptor

## Abstract

**Background:**

Chronic and frequently recurring infectious diseases, such as malaria, are associated with expanded populations of atypical memory B cells (MBCs). These cells are different from classical MBCs by the lack of surface markers CD21 and CD27 and increased expression of inhibitory receptors, such as FcRL5. While the phenotype and conditions leading to neogenesis of atypical MBCs in malaria-experienced individuals have been studied extensively, the origin of these cells remains equivocal. Functional similarities between FcRL5^+^ atypical MBCs and FcRL5^+^ classical MBCs have been reported, suggesting that these cells may be developmentally related.

**Methods:**

Here, a longitudinal analysis of FcRL5 expression in various B cell subsets was performed in two children from a high transmission region in Uganda over a 6-month period in which both children experienced a malaria episode. Using B-cell receptor (BCR)-sequencing to track clonally related cells, the connections between IgM^+^ and IgG^+^ atypical MBCs and other B cell subsets were studied.

**Results:**

The highest expression of FcRL5 was found among IgG^+^ atypical MBCs, but FcRL5^+^ cells were present in all MBC subsets. Following malaria, FcRL5 expression increased in all IgM^+^ MBC subsets analysed here: classical, activated, and atypical MBCs, while results for IgG^+^ MBC subsets were inconclusive. IgM^+^ atypical MBCs showed few connections with other B cell subsets, higher turnover than IgG^+^ atypical MBCs, and were predominantly derived from naïve B cells and FcRL5^−^ IgM^+^ classical MBCs. In contrast, IgG^+^ atypical MBCs were clonally expanded and connected with classical MBCs. IgG^+^ atypical MBCs present after a malaria episode mainly originated from FcRL5^+^ IgG^+^ classical MBCs.

**Conclusions:**

Collectively, these results suggest fundamental differences between unswitched and class-switched B cell populations and provide clues about the primary developmental pathways of atypical MBCs in malaria-experienced individuals.

**Supplementary Information:**

The online version contains supplementary material available at 10.1186/s12936-021-03970-1.

## Background

Malaria is a potentially deadly disease that mainly impacts people living in Sub-Saharan Africa and Southeast Asia. In 2019, an estimated 229 million malaria cases and over 400,000 malaria deaths were reported, with the highest rates of morbidity and mortality among children under the age of five [1]. The disease is caused by parasites of the *Plasmodium* genus, of which *Plasmodium falciparum* is the most common and deadliest species [1]. People in endemic regions develop immunity to malaria over the course of years of recurrent infections [2, 3]. An important component of immunity against disease is an IgG antibody response that controls parasitaemia during the blood stage, resulting in asymptomatic infections [4–8]. In the past decade, it has become clear that *Plasmodium* infections shape the memory B cell (MBC) compartment, leading to an accumulation of atypical MBCs [9, 10]. The role of atypical MBCs in the protective B cell response to *P. falciparum* is incompletely understood [11]. The accumulation of atypical MBCs appears to be driven by a combination of prolonged antigen exposure and cytokine stimulation of B cells within the highly inflammatory environment of *P. falciparum* infections [12, 13]. While the conditions leading to the generation of atypical MBCs are largely known, the origin of atypical MBCs and their connections to other B cell populations remain to be established. A deeper understanding of the developmental pathways of atypical MBCs may provide clues about the function of these cells in the B cell response to *P. falciparum* infections.

Atypical MBCs lack expression of MBC markers CD21 and CD27, and typically express T-bet, CXCR3, and CD11c, as well as inhibitory markers, including FcRL3 and FcRL5 [10, 14–16]. Atypical MBCs cells are not unique to *Plasmodium* infections and have also been identified in other chronic inflammatory conditions, such as HIV infection and systemic lupus erythematosus [17, 18]. High expression of inhibitory receptors and failure to activate these cells in in vitro cultures containing soluble antigens and inflammatory cytokines initially suggested that these cells are dysfunctional and do not contribute to immunity against infection [15–17]. However, there is now increasing evidence that supports a functional role of these cells in humoral immune responses. For example, atypical MBCs in malaria-experienced individuals contained secretory immunoglobulin transcripts, suggesting that these are antibody-secreting cells, while classical CD21^+^CD27^+^ MBCs in the same individuals did not [19]. In addition, atypical MBCs responded robustly to membrane-bound antigens, resulting in upregulation of mRNA encoding the transcription factor IRF4, which is essential for differentiation into antibody-secreting cells [20]. Finally, atypical MBCs were found in small numbers in healthy individuals and were shown to be part of a normal response to infection and immunization [21, 22].

**Fig. 1:**
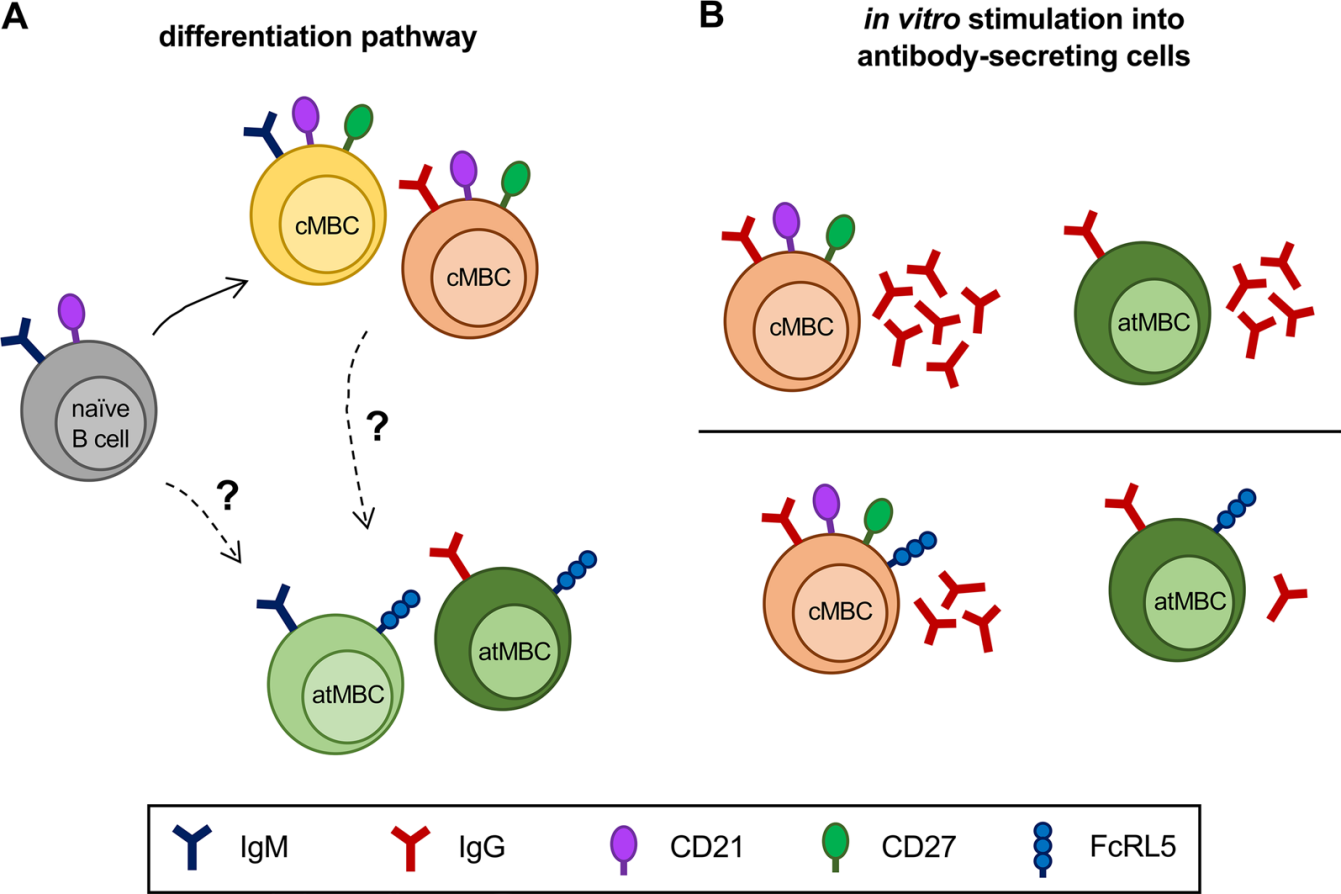
Summary of the current knowledge on the origin of and connections between classical and atypical MBCs. **A** Potential differentiation pathways of classical memory B cells (cMBCs) and atypical memory B cells (atMBCs). Upon antigen exposure, naïve B cells become activated and can differentiate into antibody-secreting cells (not shown) or classical MBCs. Atypical MBCs are detected after primary *P. falciparum* infection and may thus be derived from naïve B cells, either directly or through an intermediate cell type. It is not known whether classical MBCs can also differentiate into atypical MBCs after antigen exposure, and whether there is a difference in differentiation pathways between IgM^+^ and IgG^+^ atypical MBCs. Classical MBCs are defined by the expression of surface markers CD21 and CD27, while the majority of classical MBCs lack FcRL5^−^. Atypical MBCs are defined by the absence of CD21 and CD27. Most atypical MBCs express FcRL5 and other markers described in the main text. **B** FcRL5^−^ and FcRL5^+^ classical and atypical MBCs showed differences in their ability to differentiate into antibody-secreting cells when stimulated in vitro with soluble antigen and other stimuli. The relative number of antibody-secreting cells after stimulation is represented by the number of immunoglobulin molecules. These results suggest that FcRL5^+^ classical and atypical MBCs may be developmentally related

Upon immunization, the atypical MBC pool expands transiently, peaking 4 weeks post-antigen exposure, followed by a gradual decline over the course of several months back to pre-vaccination levels [21]. Similarly, the atypical MBC population also expands shortly after a malaria episode and contracts slowly over the course of several months [14]. Expansion of this cell population was observed in individuals with primary *P. falciparum* infection, suggesting that atypical MBCs could originate from naïve B cells [14]. This would be in line with in vitro studies showing that naïve B cells can differentiate into atypical MBCs under specific culture conditions [23, 24]. However, malaria-experienced individuals showed a greater increase in atypical MBC frequency after a malaria episode than individuals with a primary infection [14], suggesting that atypical MBCs may also be derived from antigen-experienced B cells, presumably resting classical MBCs. In the above-mentioned studies, no distinction was made between unswitched and class-switched B cells, and the differentiation pathways of atypical MBCs with different isotypes remain largely uncharacterized (Fig. 1A).

Prior observations by Sullivan et al*.* also suggested there may be connections between atypical and classical MBCs, since both atypical and classical IgG^+^ MBCs contained FcRL5^−^ and FcRL5^+^ subpopulations and the surface expression of FcRL5 in both atypical and classical subsets was associated with higher levels of FcRL3, CD19, and CD20 expression [16]. Furthermore, FcRL5^+^ classical MBCs showed lower rates of differentiation into antibody-secreting cells upon stimulation than their FcRL5^−^ counterparts, and a similar difference was observed between FcRL5^−^ and FcRL5^+^ atypical MBCs [16] (Fig. 1B). These phenotypic and functional similarities between FcRL5^+^ classical and atypical MBCs led us to speculate that these subsets may be developmentally related.

One approach to gain insight into the developmental pathways of B cells is to analyse clonally related cells with shared B cell receptor (BCR) sequences. Since almost every BCR is unique, these sequences can serve as barcodes to identify B cells that originated from the same precursor or to track their differentiation over time. Using this methodology, it was previously observed that the level of connectivity between classical and atypical MBCs was similar for unswitched and switched subsets in malaria-experienced adults [15]. In contrast, a second study detected no overlap in BCR sequences among IgG^+^ classical and atypical MBCs specific for two *P. falciparum* merozoite antigens in malaria-experienced adults [19]. These conflicting findings highlight the need to further study the connections between these B cell subsets. Here, clonal B cell analysis was used to better understand the origin and dynamics of IgM^+^ and IgG^+^ atypical MBCs in two 5-year old children who resided in an endemic region in Uganda. Using longitudinal samples that covered a malaria-free period and a period in which these children experienced malaria, malaria-induced changes in clonal expansion within and clonal connections between MBC subsets were assessed. Flow cytometric analysis revealed differences in FcRL5 expression between unswitched and class-switched B cells in atypical and activated MBC populations. BCR-sequencing analysis of sorted MBC populations showed that IgM^+^ atypical MBCs had few clonal connections with other memory B cell subsets, had a high turnover rate, and appeared to be mostly derived from naïve B cells and IgM^+^ FcRL5^−^ classical MBCs. IgG^+^ atypical MBCs, on the other hand, were maintained longer and appeared to predominantly originate from IgG^+^ FcRL5^+^ classical MBCs.

## Methods

### Isolation of B cells

Cryopreserved PBMCs from two children of the PRISM cohort were rapidly thawed and stained on ice for 30 min with an antibody panel against lymphocyte markers (Additional file 1: Table S1). B cells were defined as CD3^−^CD14^−^CD19^+^CD20^+^ and sorted by flow cytometry into the following subsets: naïve B cells (CD21^+^, CD27^−^, IgM^+^, and IgG^−^), FcRL5^−^ classical MBCs (CD21^+^, CD27^+^, FcRL5^−^, and IgG^+^ or IgM^+^), FcRL5^+^ classical MBCs (CD21^+^, CD27^+^, FcRL5^+^, and IgG^+^ or IgM^+^), and FcRL5^+^ atypical MBCs (CD21^−^, CD27^−^, FcRL5^+^, and IgG^+^ or IgM^+^). Cells were sorted directly into ice-cold lysis buffer and subsequently used for BCR sequencing.

### BCR-sequencing

Total RNA was extracted from sorted B cell subsets using Qiagen’s RNeasy Micro Kit and reverse transcribed using the iScript cDNA Synthesis Kit (Bio-Rad). The cDNA was used as template for IgM-VH and IgG-VH amplification by PCR (Advantage 2 PCR Kit, Takara Bio) using a pool of custom-designed IGHV1-7 family forward primers and IgG- or IgM-specific reverse primers containing IonXpress barcodes to uniquely tag each subset/isotype prior to library prep (Additional file 1: Table S2). Amplicons were separated using a 1.5% agarose gel, stained with Sybr Safe and extracted from gel using Qiagen’s MiniElute Kit. Gel-purified DNA was quantified (High Sensitivity DNA Kit and Bioanalyzer, Agilent Biotechnologies) and subsequently diluted to ~ 15 pM to create an equimolar DNA library. An emulsion PCR (Ion OneTouch2 Kit, Life Technologies) was used to bind and clonally expand DNA fragments onto IonSphere beads for sequencing. Quality of the DNA-enriched beads was checked using a Qubit Fluorometer (Thermo) and subsequently sequenced on an Ion PGM System sequencer using 318 v2 chips (Life Technologies). Fastq sequence files were generated based on raw sequencing output files (BAM format) retrieved from the IonTorrent PGM Sequencer. In general, next-generation sequencing technologies are error-prone [25], which, in the case of Ig-VH repertoire sequencing, can lead to overestimation of SHM and Ig repertoire diversity. A bioinformatics pipeline was applied to first discard nonproductive (i.e., incorrect reading frame) sequencing reads. For all reads, IGHV and IGHJ usage, and HCDR3 amino acid sequence were determined using a custom pipeline incorporating sequence alignments and MiXCR [26]. The compiled bioinformatics pipeline is available for download at https://github.com/swuecho/Immune-repertoire-network. This information, along with the B cell subset of origin and isotype, was used to annotate each productive Ig-VH read. Unique sequences that were not supported by at least one other sequence read were discarded to further reduce overestimation of diversity due to errors introduced during PCR amplification or sequencing. Finally, sequences with identical IGHV and IGHJ usage and HCDR3 amino acid sequence in each B cell subset were collapsed into a single sequence. Processed data files containing IGHV and IGHJ calls and HCDR3 sequences are available for download at https://github.com/embunnik/clonal_scores.

### Clonality scores

Clonal expansion and clonal connection scores were calculated within and between each B cell subsets (e.g., IgM^+^FcRL5^−^ classical MBCs or IgG^+^ atypical MBCs) using a custom Perl script available for download at https://github.com/embunnik/clonal_scores. Sequences were first grouped into a clonotype when the following criteria were met: same IGHV gene usage, same IGHJ gene usage, same HCDR3 length, and at least 85% amino acid sequence identity in HCDR3 [27, 28]. The clonal expansion score within each B cell subset was then calculated as the percentage of reads in all clonotypes out of the total number of sequences in the population. For example, if a population of 9 sequences has two sequences that belong to clonotype 1, three sequences that belong to clonotype 2, and four sequences that are unique, the clonality score is (2 + 3)/9 = 56% (Additional file 1: Fig. S1). For the calculation of clonal connection scores between two B cell subsets, the percentage of clonotype reads present in both subsets was calculated separately for each population (Additional file 1: Fig. S1). The clonality score of the smallest population was used for analysis, based on the rationale that the size of the smaller B cell population would be limiting for the number of shared clonotypes detected in both populations (Additional file 1: Fig. S1).

### Morisita-Horn similarity indexes

Morisita-Horn similarity indexes were calculated based on the IGHV gene usage, IGHJ gene usage, and HCDR3 length according to the formula of overlap published by Horn [29]:

$$C_{{\text{H}}} = \frac{{2\mathop \sum \nolimits_{i = 1}^{S} x_{i} y_{i} }}{{\left( {\frac{{\mathop \sum \nolimits_{i = 1}^{S} x_{i}^{2} }}{{X^{2} }} + \frac{{\mathop \sum \nolimits_{i = 1}^{S} y_{1}^{2} }}{{Y^{2} }}} \right) XY}}$$

in which *x*_*i*_ is the number of times the combination of IGHV gene, IGHJ gene and CDRH3 length *i* is present in one sample of size *X*; and *y*_*i*_ is the number of times that same combination of IGHV gene, IGHJ gene and CDRH3 length is present in a second sample of size *Y*.

### Visualization and statistics

All graphs were generated in RStudio (v1.4.1103) using R v4.0.4. Boxplots and dotplots were plotted using the package ggplot2. In all boxplots throughout the article, the center line indicates the median, upper, and lower limits of the box indicate the first and third quartiles, and the whiskers indicate the upper and lower limits of the data within 1.5 times the interquartile range. Heatmaps were generated using the package pheatmap. All statistical tests were performed using the non-parametric paired Wilcoxon signed-rank test, using matching data from the same time point or time period and child. Of note, for groups of 6 data points, the smallest possible P value using this test is 0.03.

## Results

### Longitudinal study setup

**Fig. 2:**
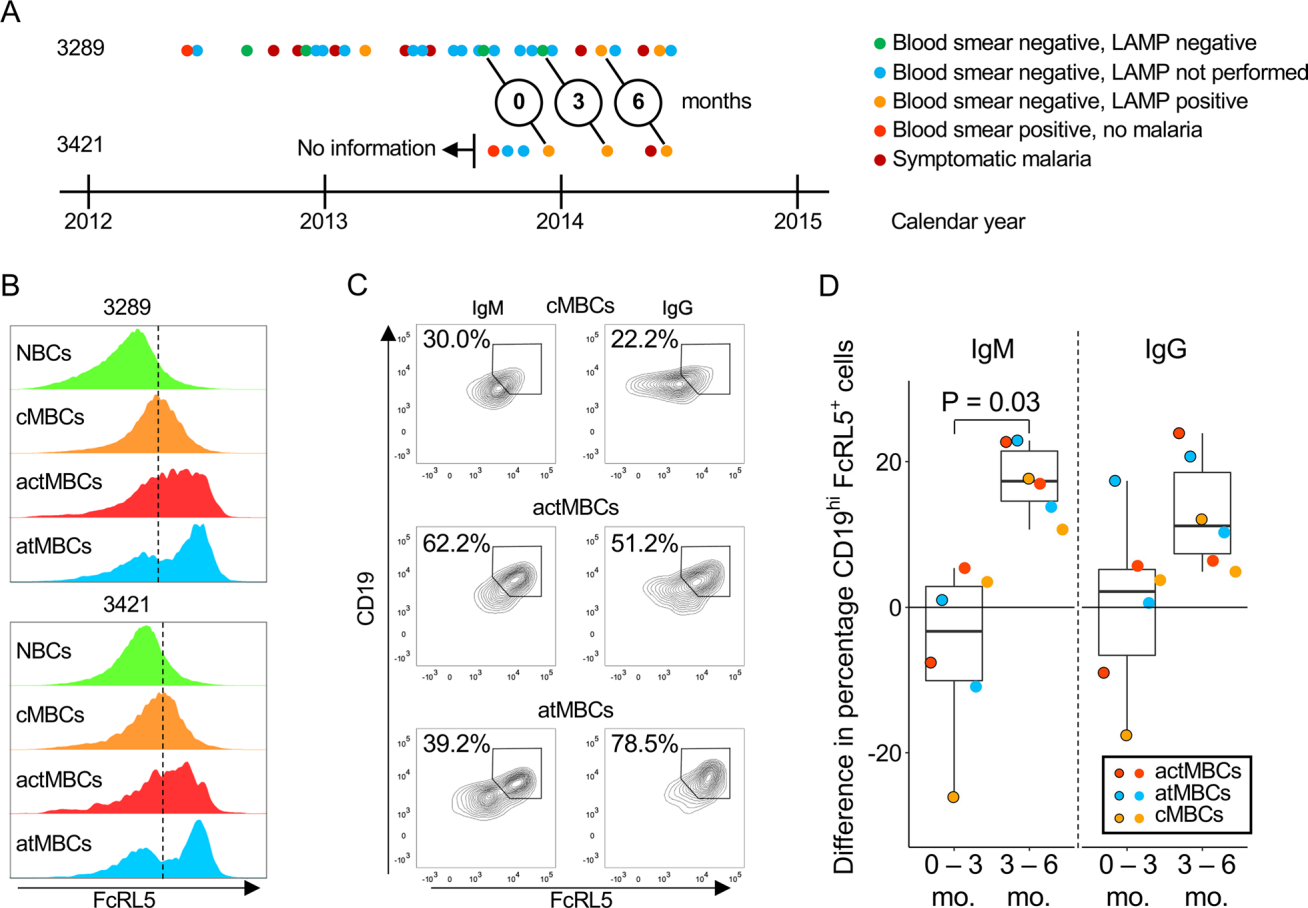
Changes in FcRL5 expression in different B cell subsets after malaria. **A** Schematic overview of the medical history of the two 5-year old children included in this study. The three time points of sampling are indicated (circles with 0, 3, and 6 months). Both children had a positive blood smear without malaria in the 1.5 years prior to the first time point and an episode of uncomplicated malaria between the second and the third time point. **B** Expression of FcRL5 on the cell surface of naïve B cells (NBCs; CD21^+^CD27^−^), classical memory B cells (cMBCs, CD21^+^CD27^+^), activated memory B cells (actMBCs; CD21^−^CD27^+^), and atypical memory B cells (atMBCs; CD21^−^CD27^−^) after malaria. **C** The percentage of cells that are CD19^hi^FcRL5^+^ among unswitched (IgM^+^) and class-switched (IgG^+^) classical MBCs, activated MBCs, and atypical MBCs. **D** Changes in the percentage of CD19^hi^FcRL5^+^ cells among IgM^+^ and IgG^+^ classical MBCs, activated MBCs, and atypical MBCs during the malaria-free period (time points at 0 and 3 months) and the period with a malaria episode (3 to 6 months). Data points with a black outline show results for child with cohort ID 3289, while data points without an outline depict results for child with cohort ID 3421

To study the effect of a malaria episode on FcRL5 expression in various B cell subsets and the clonal connections within and between these subsets, two 5-year old children who were included in the Program for Resistance, Immunology, Surveillance and Modeling of Malaria (PRISM) cohort study in the high-transmission region of Tororo, Uganda (cohort IDs: 3289 and 3421) were selected [30]. Although information about prior medical history is largely lacking for the child with cohort ID 3421 (Fig. 2A), children in this region typically experience multiple malaria episodes before age five. In the 1.5 years prior to sample collection, both children showed evidence of a developing immune response against *P. falciparum* in the form of a positive blood smear in the absence of malaria, followed by parasite clearance without treatment. In the PRISM cohort, cryopreserved peripheral blood mononuclear cells (PBMCs) were routinely obtained from participants at 3-month intervals. For each child, three sequential time points covering a total of 6 months were selected, with an episode of uncomplicated malaria 3 to 4 weeks prior to the third time point (Fig. 2A). This sampling scheme allowed us to compare B cell dynamics between a malaria-free period (0 months to 3 months) and a period in which the children had malaria (3 months to 6 months). During the malaria-free period, it would be expected to observe either no changes to the B cell compartment or a reversion of malaria-induced changes from prior episodes, while malaria-induced changes were expected between 3 and 6 months. The child with cohort ID 3421 had sub-patent parasitaemia at all time points, while the child with cohort ID 3289 had no detectable parasitaemia at the first two time points and sub-patent parasitaemia on the third time point. While the effect of low-level parasitaemia on the immune response is not fully understood, it was recently reported that asymptomatic *P. falciparum* infections during the dry season result in minimal immune activation [31]. It is, therefore, likely that changes in B cells responses induced by massive immune activation during malaria will be different as compared to those during asymptomatic infection.

### Increased FcRL5 expression in IgM^+^ memory B cell subsets after malaria

Previously, it was reported that classical and atypical MBC populations in malaria-experienced individuals were heterogeneous in FcRL5 expression and showed related functional differences [16]. Here, the impact of a malaria episode on the expression of FcRL5 on the cell surface of naïve B cells (CD21^+^CD27^−^), classical MBCs (CD21^+^CD27^+^), activated MBCs (CD21^−^CD27^+^), and atypical MBCs (CD21^−^CD27^−^) was evaluated by flow cytometry (Fig. 2B, Additional file 1: Figs. S2–S4). To exclude plasmablasts and plasma cells from this analysis, B cells were first gated as CD19^+^CD20^+^. In both children, a large proportion of activated and atypical MBCs expressed FcRL5, with a range of expression levels in activated MBCs and more clearly distinguished FcRL5^−^ and FcRL5^+^ subpopulations in atypical MBCs (Fig. 2B). In contrast, FcRL5 expression was low in classical MBCs and almost completely absent in naïve B cells (Fig. 2B). FcRL5 expression on atypical MBCs is not the only characteristic of an atypical B cell phenotype and is often accompanied by altered expression of other surface markers, including high expression of the universal B cell marker CD19 [16]. This raises the question whether FcRL5 expression is also linked to high CD19 expression on other B cell populations. Similar to atypical MBCs, the large majority of FcRL5^+^ cells among classical and activated MBCs also expressed high levels of CD19 on their surface (Fig. 2C). Subsequently, differences between unswitched (IgM^+^) and class-switched (IgG^+^) cells within each B cell subset were analysed. While no clear differences were seen between IgM^+^ and IgG^+^ classical and activated MBCs, the fraction of FcRL5^+^CD19^hi^ atypical MBCs was approximately twice as large for the IgG^+^ cells when compared to the IgM^+^ subset (Fig. 2C, Additional file 1: Figs. S3, S4). Of all B cell populations, atypical MBCs contained the largest fraction of IgG^+^ CD19^hi^ FcRL5^+^ cells (average, 65% versus 15% and 41% for classical and activated MBCs, respectively), while activated MBCs had a higher frequency of IgM^+^ CD19^hi^ FcRL5^+^ cells (average, 40%) as compared to classical and atypical MBCs (average, 21% and 20%, respectively, Fig. 2C, Additional file 1: Figs. S3, S4).

During the malaria-free period (0 months to 3 months), the percentage of IgM^+^ FcRL5^+^ cells decreased or remained stable, both when calculated relative to all IgM^+^ B cells or MBC subsets (Additional file 1: Fig. S5) and in each MBC subset separately (median, − 3.3%; range, − 26–5%; Fig. 2D). In contrast, IgM^+^ FcRL5^+^ cells increased in frequency after the malaria episode (median, 17%; range, 11–23%; Fig. 2D, Additional file 1: Fig. S5), suggesting that malaria may drive FcRL5 expression in all IgM^+^ MBCs. The percentage of FcRL5^+^ cells also increased after malaria among IgG^+^ B cells (median, 11%; range, 5–24%; Fig. 2D, Additional file 1: Fig. S5). However, some of the IgG^+^ MBC subsets also showed an increase in FcRL5 expression during the malaria-free period (Fig. 2D), and it can therefore not be concluded that the increase in FcRL5 expression in these B cell subsets was caused by the malaria episode.

In summary, in these two children, the highest expression of FcRL5 was found among IgG^+^ atypical MBCs, but FcRL5^+^ cells were present in all MBC subsets. Malaria appeared to induce the expression of FcRL5 on all IgM^+^ MBC subsets analysed here. While IgG^+^ MBC subsets followed the same trend, results were inconclusive, possibly pointing to differences in the dynamics between IgM^+^ and IgG^+^ MBC subsets in response to infections. Differences in FcRL5 expression between IgM^+^ and IgG^+^ MBCs underscore the notion that these subsets are distinct and highlights the importance of analysing these cells separately.

### Generation and analysis of longitudinal B cell receptor sequencing data

To further investigate the relationship between different B cell populations, naïve B cells, FcRL5^−^ and FCRL5^+^ classical MBCs, and FcRL5^+^ atypical MBCs were isolated from these two children using fluorescence-activated cell sorting. BCR-sequencing was then performed on the sorted cells to study how clonal expansion within and clonal connections between these B cell populations were affected by malaria. Unfortunately, relatively low numbers of activated MBCs were isolated from some samples (< 500 cells) and this population was therefore excluded from the study as it would not be possible to achieve sufficient sequencing depth (Additional file 1: Table S3). Heavy chain variable region sequence reads were bioinformatically split by antibody isotype (IgM or IgG) and analysed for IGHV and IGHJ gene segment usage. To correct for over- or underrepresentation of sequences due to PCR amplification biases and to prevent overestimating the level of clonal expansion, sequences with identical IGHV and IGHJ usage and heavy chain complementary-determining region 3 (HCDR3) sequence within each subset, which could theoretically all originate from the same single B cell, were collapsed into a single sequence (Additional file 1: Table S4).

**Fig. 3:**
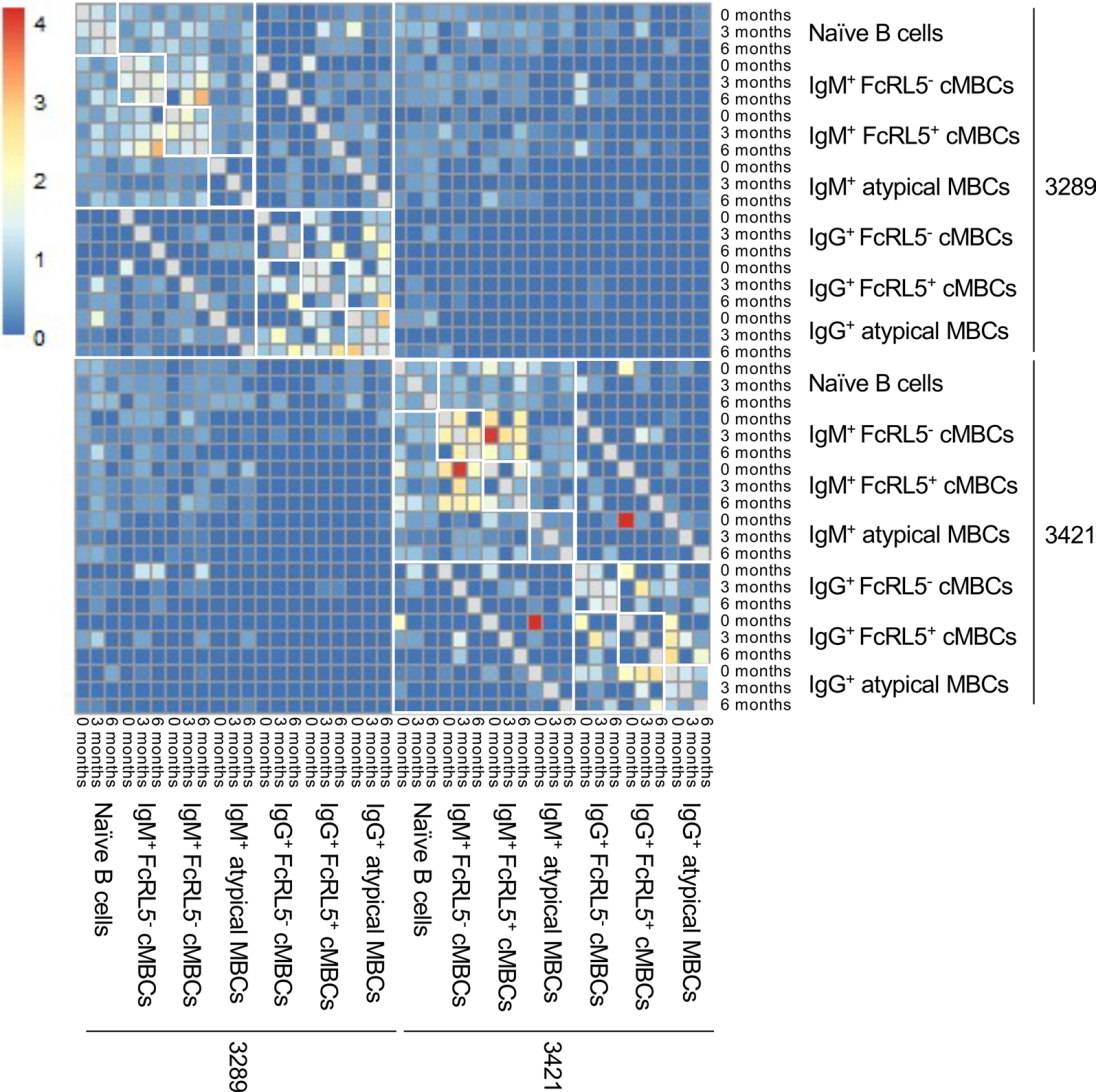
All-against-all heatmap of clonal connection scores between all B cell subsets for both children. Clonal connection scores range between 0% (lowest in blue) and 4% (highest in red). The highest clonal connection scores are seen within IgM^+^ and IgG^+^ subsets of each child, indicating that samples have not been mixed. To be able to visualize low clonal connection scores between donors, the clonal expansion scores (within B cell populations) have been removed

Next, clonality scores were calculated within and between B cell subsets of the same isotype. The clonality score calculated *within* a subset (e.g., IgG^+^ atypical MBCs) represents the amount of *clonal expansion*, which is a measure of cell division and selection upon B cell activation. The clonality score *between* B cell subsets (e.g., FcRL5^+^ IgM^+^ classical MBCs and IgM^+^ atypical MBCs) is a measure of *clonal connections*, which can be the result of both B cell subsets having the same precursor or from direct differentiation of B cells from one subset into another subset. B cells were considered clonally related when their BCR sequences contained the same IGHV and IGHJ genes and had at least 85% similarity in the HCDR3 amino acid sequence [15, 27, 28, 32]. Clonality scores were expressed as the percentage of sequences for which at least one clonally related sequence was identified (i.e., a clonotype; Additional file 1: Fig. S1), and were calculated for each B cell subset at each time point, as well as for each pairwise comparison between B cell subsets at the same time point and across time points (see “Methods”). The PBMCs used for this study were obtained from a few milliliters of blood and the sequencing data set thus represents a small portion of the total B cell repertoire. As a result, clonal connection scores between B cell subsets are small and clonotypes typically consisted of only two sequences, similar to results presented in [33]. To evaluate whether such sparse connections could be the result of random similarities in BCR sequences and to confirm sample identity, an all-against-all heatmap of these clonality scores was generated (Fig. 3). This plot shows separation of samples from the two children. At ≥ 85% HCDR3 similarity, there were low levels of connections between the IgM^+^ compartments of both children. However, clonal connections within IgM^+^ and IgG^+^ compartments of the same child were more abundant and larger in magnitude, suggesting that this analysis detects true connections between B cell subsets. In all analyses except those that investigate the origin of atypical MBCs, naïve B cells serve as a control to establish a baseline of the clonal expansion and connections scores in this data set.

### IgM^+^ atypical MBCs have low levels of clonal expansion and high levels of turnover

**Fig. 4:**
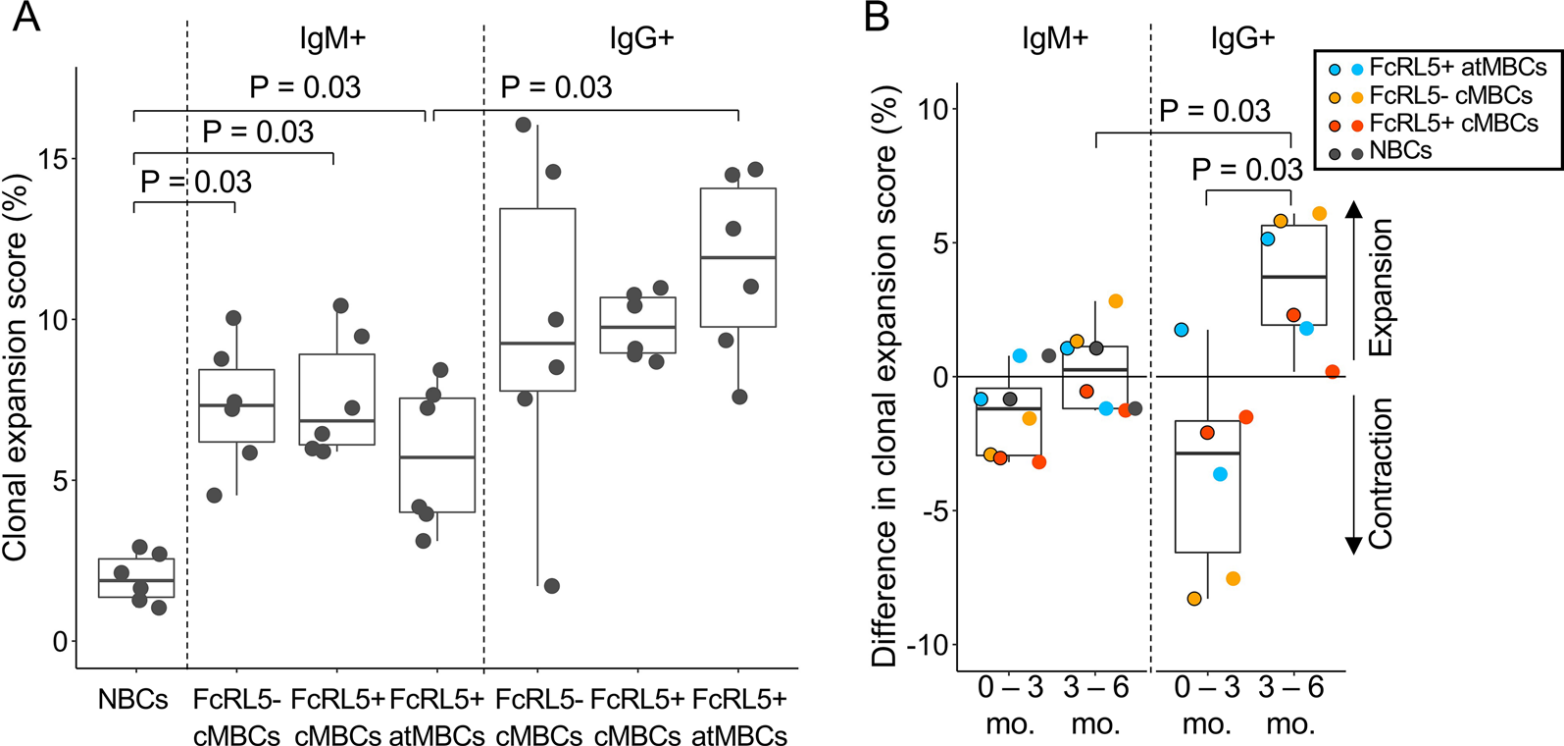
Larger clonal expansion in IgG^+^ B cell subsets after malaria. **A** IgG^+^ atypical MBCs show larger expansion than IgM^+^ atypical MBCs at all time points. Each data point represents the clonal expansion score at one time point in one child. **B** The difference in clonal expansion scores between two consecutive time points, with the malaria-free period (0 to 3 months) on the left and the period with a malaria episode (3 to 6 months) on the right for both IgM^+^ and IgG^+^ B cell subsets. Data points with a black outline show results for child with cohort ID 3289, while data points without an outline depict results for child with cohort ID 3421. In both panels, the non-parametric paired Wilcoxon signed-rank test was used to test for statistically significant differences between B cell subsets. *NBCs* naïve B cells, *cMBCs* classical memory B cells, *atMBCs* atypical memory B cells, *mo.* months

First, the level of clonal expansion within each of the described B cell subsets at each time point was evaluated. As expected, clonal expansion scores among naïve B cells were relatively low (range, 1–3%, Fig. 4A). For both FcRL5^−^ and FcRL5^+^ classical MBCs, clonal expansion scores ranged between 4 and 16%, and were not different between IgM^+^ and IgG^+^ subsets (Fig. 4A). The clonal expansion scores were similar for atypical MBCs as compared to classical MBCs (range, 3–15%). However, IgM^+^ atypical MBCs showed significantly lower clonal expansion than IgG^+^ atypical MBCs at all three time points for both children (P = 0.03, Wilcoxon signed-rank test; Fig. 4A). Over the course of the malaria-free period, nearly all B cell subsets contracted (data points below the null line), without a distinct difference between IgM^+^ and IgG^+^ cells (Fig. 4B). Five out of six IgG^+^ subsets showed expansion after the malaria episode, while one showed no expansion or contraction, and this expansion was always larger than for the corresponding IgM^+^ populations (P = 0.03, Wilcoxon signed-rank test; Fig. 4B). In both children, FcRL5^−^ IgG^+^ classical MBCs showed the largest changes over time, both in contraction during the malaria-free period (− 8%) and in expansion after a malaria episode (+ 6%; Fig. 4B) as compared to FcRL5^+^ IgG^+^ classical MBCs and IgG^+^ atypical MBCs. These results might indicate that out of these three MBC subsets, FcRL5^−^ IgG^+^ classical MBCs are most responsive to *Plasmodium* infection.

**Fig. 5:**
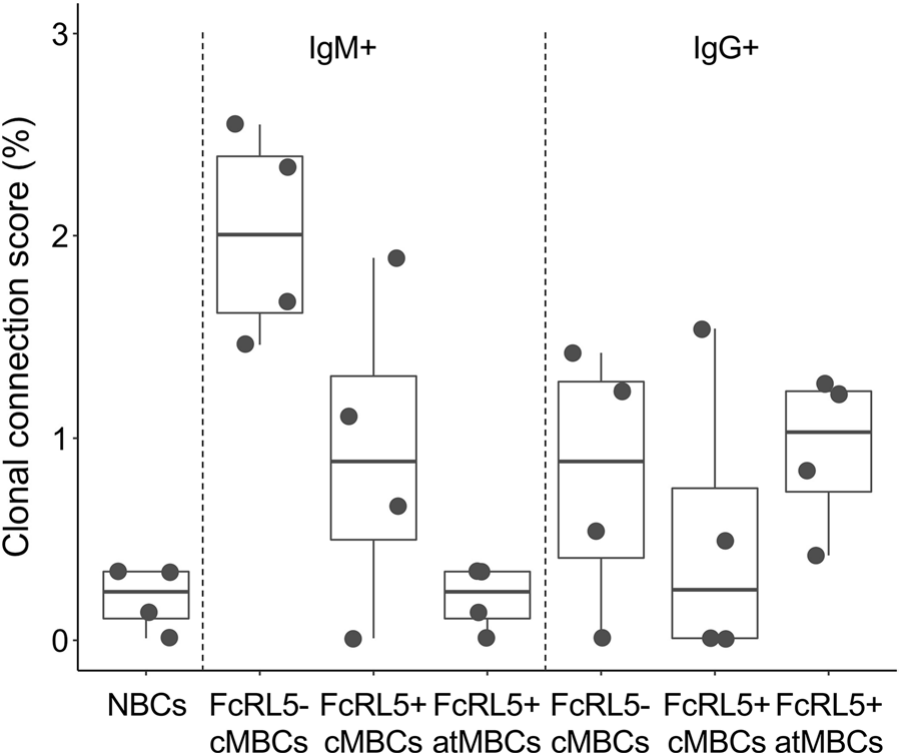
IgM^+^ atypical MBCs show higher turnover than other B cell subsets. Clonal connection scores were calculated within the same B cell subset between two subsequent time points and plotted as dots. *NBCs* naïve B cells, *cMBCs* classical memory B cells, *atMBCs* atypical memory B cells

Next, it was questioned whether B cells present at one time point could still be detected at the following time point or whether there was turnover within B cell subsets from one time point to the next. To estimate the turnover of B cells in each subset, the clonal connection scores between the same B cell subsets over the course of both 3-month intervals were analysed. If no connections are found within the same B cell subset at two subsequent time points, this indicates that all B cells present at the first time point have disappeared from the circulation and have been replaced by other cells. Since this analysis has only four data points in each group, statistically significant differences between groups were not evaluated, but only the most prominent observations were described. IgM^+^ atypical MBCs showed the same low level of connections between time points as naïve B cells (range: 0–0.3%), and always showed fewer connections than FcRL5^−^ IgM^+^ classical MBCs (range: 1.5–2.6%; Fig. 5). With one exception, the same was true for the comparison between IgM^+^ atypical MBCs and FcRL5^+^ IgM^+^ classical MBCs, although the difference was smaller. In contrast, no difference in turnover was observed between IgG^+^ atypical MBCs and both FcRL5^−^ and FcRL5^+^ classical MBCs. While reduced connections of IgM^+^ atypical MBCs between time points may partially be explained by their lower levels of clonal expansion, it might also point towards an increased disappearance of IgM^+^ atypical MBCs from the circulation. Of note, there was no consistent pattern between the clonal connection scores within B cell subsets over 3-month intervals in the absence or presence of a malaria episode.

Collectively, these data suggest that IgM^+^ atypical MBCs are distinct in their dynamics as compared to other MBC subsets, showing lower levels of clonal expansion and higher levels of turnover. These results provide further support for the idea that IgM^+^ and IgG^+^ atypical MBCs may have different developmental pathways.

### IgM^+^ and IgG^+^ atypical MBCs may have different origins

**Fig. 6:**
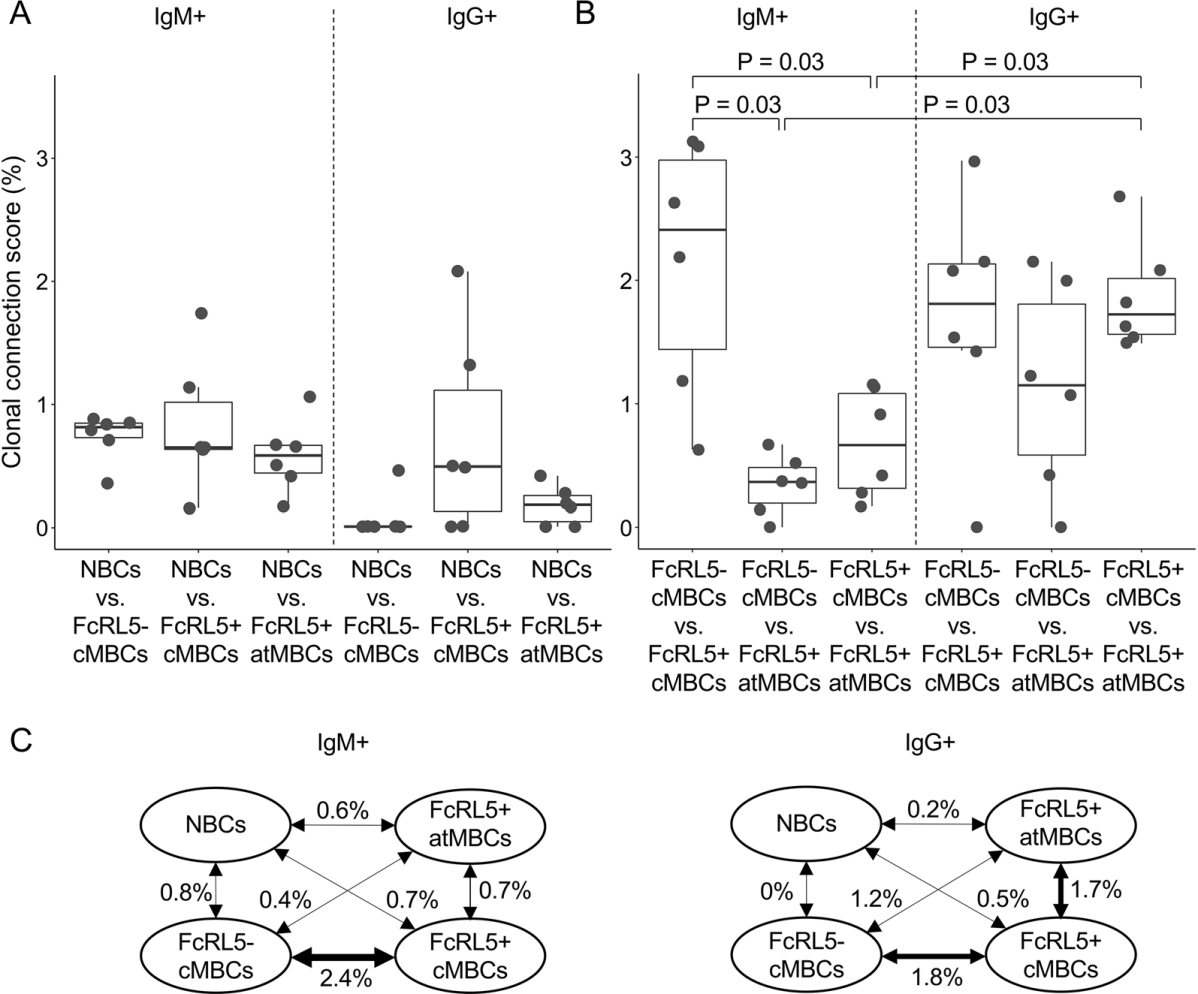
Fewer clonal connections between IgM^+^ atypical MBCs and IgM^+^ classical MBC subsets. **A** Clonal connection scores for pairs of naïve B cells and other B cell subsets. **B** Clonal connection scores for pairs of memory B cell subsets. In panels A and B, clonal connection scores between pairs of B cell subsets were calculated separately for each time point and each child and presented as dots. The non-parametric paired Wilcoxon signed-rank test was used to test for statically significant differences in connections between pairs of B cell subsets. **C** Schematic representation of the median clonal connection scores between each pair of B cell subsets. *NBCs* naïve B cells, *vs* versus, *cMBCs* classical memory B cells, *atMBCs* atypical memory B cells

Thus far, only connections *within* memory B cell subsets have been evaluated. To analyse the relationships *between* memory B cell subsets, clonal connections between different subsets that were present at the same time point were determined. The median clonal connection scores between NBCs and other B cell subsets were below 1% (Fig. 6A). The connections between IgM^+^ classical MBCs and IgM^+^ atypical MBCs were in the same range, while FcRL5^−^ and FcRL5^+^ IgM^+^ classical MBCs showed more connections between each other (median 2.4%; P = 0.03 for both comparisons with IgM^+^ atypical MBCs, Wilcoxon signed-rank test, Fig. 6B). This suggests that IgM^+^ atypical MBCs form a separate compartment that is minimally connected with the IgM^+^ classical MBC subsets. While there was a large range in the clonal connection scores between FcRL5^−^ and FcRL5^+^ IgG^+^ classical MBCs and IgG^+^ atypical MBCs, there was no consistent pattern and on average, these three subsets were equally interconnected (Fig. 6B). In line with the observation that IgM^+^ atypical MBCs seem clonally distinct, higher clonal connections scores were seen between FcRL5^+^ IgG^+^ classical and atypical MBCs than between IgM^+^ classical and atypical MBCs (Fig. 6C).

**Fig. 7:**
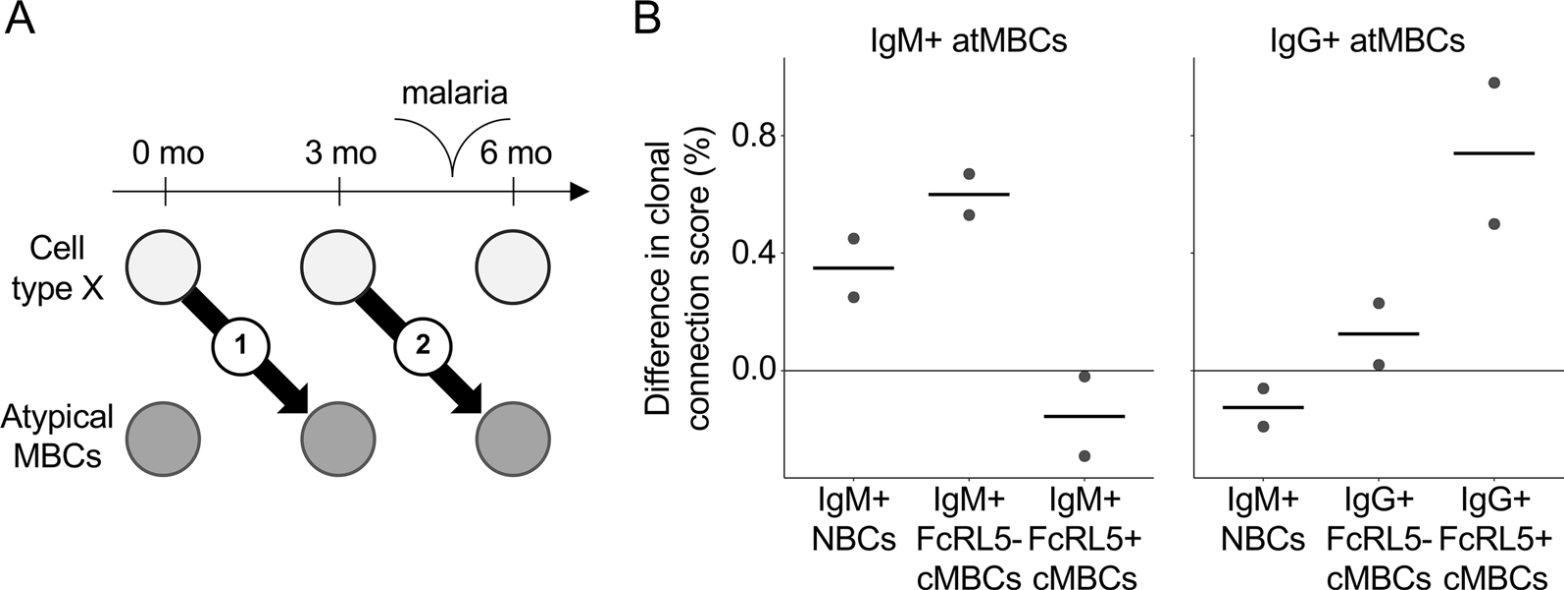
The precursors of atypical MBCs. **A** Schematic overview of the analysis and data presented in this figure. The clonal connection score for the malaria-free period (value 1) was subtracted from the clonal connection score for the period with malaria (value 2) to obtain a value that represents the net differentiation of cell type X into atypical MBCs induced by malaria. **B** The difference in clonal connections for atypical MBCs with each B cell subset mentioned on the X-axis between the period with malaria and the malaria-free period is shown as a dot for each of the two children. The red bar represents the median value. *NBCs* naïve B cells, *cMBCs* classical memory B cells, *atMBCs* atypical memory B cells

While it is known that the atypical MBC population expands after a malaria episode, it is unclear from where these cells originate. To evaluate if influx of atypical MBCs from other B cell populations contributes to their expansion after malaria, the clonal connections of B cell subsets present before the malaria episode with atypical MBCs after malaria were examined (Fig. 7A). This calculation was also performed for the connections between the same B cell subsets and atypical MBCs present before and after the malaria-free period. If a B cell subset were to differentiate into atypical MBCs as a result of malaria, it would be expected to see greater connectivity over the time period with malaria as compared to the malaria-free period. Since results from several reports suggest that atypical MBCs can be derived from naïve B cells [21, 23, 24, 32], naïve B cells were included in this analysis, in addition to the MBC subsets. This analysis only has two data points in each group and differences were therefore not tested for statistical significance. For IgM^+^ atypical MBCs, clonal connections were observed with naïve B cells, but also with FcRL5^−^ IgM^+^ classical MBCs present before the malaria episode (Fig. 7B). In contrast, these data suggest no clonal connections of IgM^+^ atypical MBCs with FcRL5^+^ IgM^+^ classical MBCs. For IgG^+^ atypical MBCs, the strongest connection was observed with pre-malaria FcRL5^+^ IgG^+^ classical MBCs (Fig. 7B). These results suggest that IgM^+^ and IgG^+^ atypical MBCs might have different precursors.

### Analysis of BCR characteristics confirms relationship between naïve B cells and IgM^+^ atypical MBCs in response to malaria

**Fig. 8:**
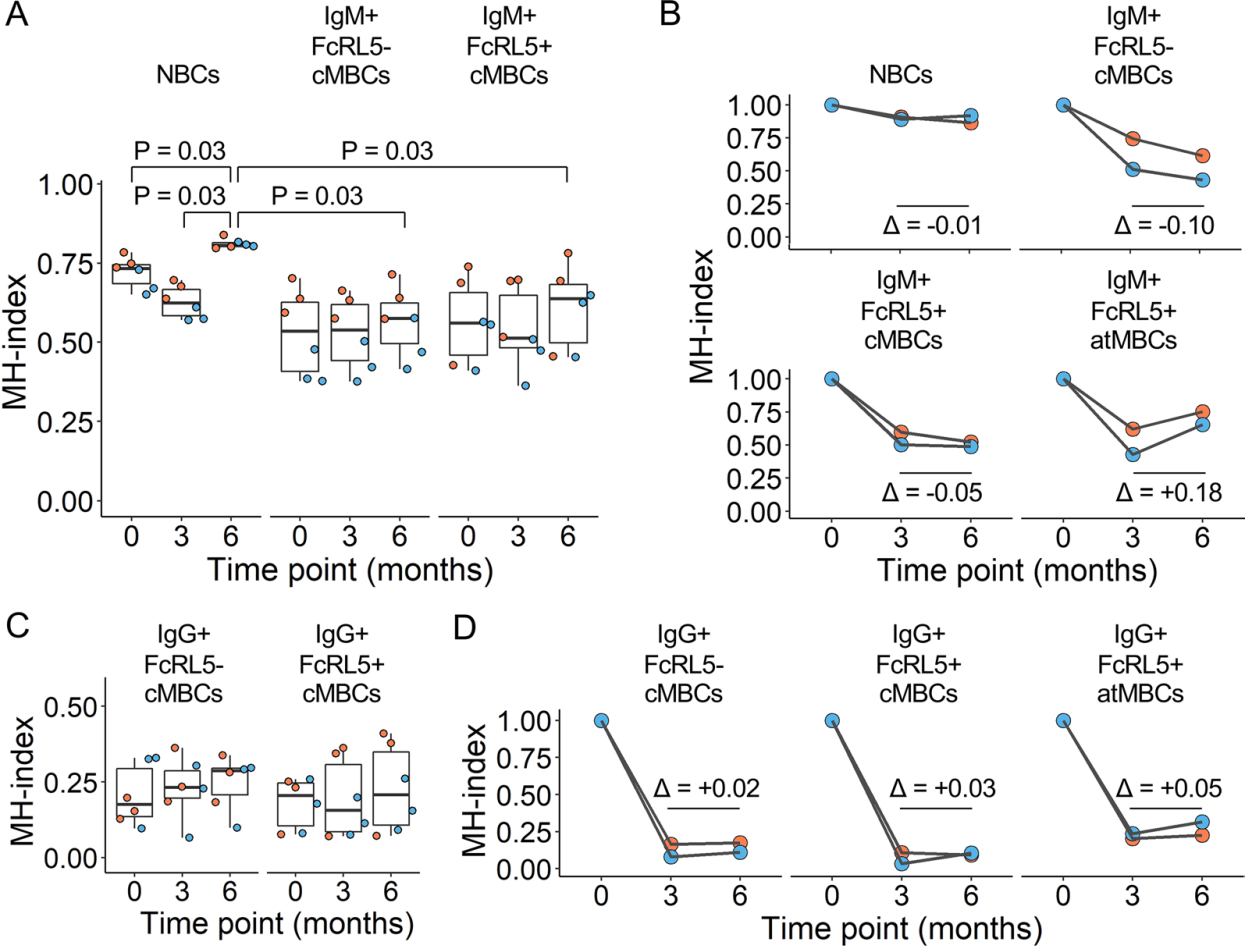
The origin of IgM^+^ atypical MBCs revealed by comparison of BCR repertoires between B cell subsets. **A** Similarity in B cell repertoires between IgM^+^ atypical MBCs at the time point indicated on the x-axis and other IgM^+^ B cell subsets as indicated above the graph at each of the three time points, resulting in three data points per child. Similarity between repertoires was calculated as the Morisita-Horn (MH) index, which ranges between a score of 0 (completely dissimilar) and 1 (identical). The non-parametric paired Wilcoxon signed-rank test was used to test for statically significant differences in MH-index between pairs of B cell subsets for both children combined. **B** Changes in BCR repertoire similarities within IgM^+^ B cell subsets. The graphs show the MH-index between cells present at the first time point and cells present at each of the three time points. The average difference in MH-index for cells at the second and third time point is indicated. **C** Similarity in B cell repertoires between IgG^+^ atypical MBCs at the time point indicated on the x-axis and other IgG^+^ B cell subsets as indicated above the graph at each of the three time points, resulting in three data points per child, as in panel **A**. **D** Changes in BCR repertoire similarities within IgG^+^ B cell subsets, as in panel **B**. Data for child with cohort ID 3289 is shown in orange, while data for child with cohort ID 3421 is shown in blue

In addition to calculating clonal connections as described above, the similarities in BCR repertoires were compared between B cell subsets, including IGHV and IGHJ usage and HCDR3 length, to investigate the origin of atypical MBCs. Since clonal connections were relatively sparse between B cell subsets, comprising at most 3.5% of all unique sequences in any given B cell subset, similarities in BCR repertoires were unlikely to be strongly influenced by these shared clonotypes. Instead, in this data set, repertoire similarities represent an independent measurement of diversity between BCR repertoires of two B cell subsets, which can be the result of differences in developmental pathways and the stimuli that drive B cell selection. To compare BCR repertoires, Morisita-Horn indexes were calculated based on IGHV and IGHJ gene usage and HCDR3 length, which provides a metric between 0 (completely dissimilar) and 1 (identical). If IgM^+^ atypical MBCs present after a malaria episode are predominantly derived from naïve B cells and FcRL5^−^ IgM^+^ classical MBCs, it would be expected to see an increase in the similarity of BCR repertoires between these populations that reflect these dynamics. Indeed, of all B cell subsets, the BCR repertoire of IgM^+^ atypical MBCs was most similar to that of the naïve B cell population (P < 0.001, Wilcoxon signed-rank test, Fig. 8A). This result suggests that IgM^+^ atypical MBCs may be activated naïve B cells that have undergone limited antigen-driven selection, since this process would result in a more diverse BCR repertoire. After malaria, the similarity between IgM^+^ atypical MBCs and naïve B cells increased, while this was not observed for the comparison between IgM^+^ classical MBCs (FcRL5^−^ or FcRL5^+^) and atypical MBCs (Fig. 8A). This observation is consistent with an influx of naïve B cells into the IgM^+^ atypical MBC compartment during or shortly after *P. falciparum* infection. The lack of an increase in similarity between IgM^+^ FcRL5^−^ classical MBCs and atypical MBCs after malaria may suggest that IgM^+^ atypical MBCs derived from classical MBCs have undergone more extensive antigen-driven selection. These results are thus not inconsistent with the analysis presented in Fig. 7, but point out differences between the two differentiation pathways. Over time, the repertoires of naïve B cells, as well as IgM^+^ FcRL5^−^ and FcRL5^+^ classical MBCs, became more diverse as compared to their corresponding populations present at the first time point, consistent with B cell turnover and influx of unrelated B cell clones (Fig. 8B). Due to the limited sample size, no tests were performed to detect statistically significant differences. The same was observed for IgM^+^ atypical MBCs after the malaria-free period (Fig. 8B). However, this decrease was followed by an increase of the Morisita-Horn index after the children had malaria (from 0.5 to 0.7; Fig. 8B). These results indicate that IgM^+^ atypical MBC responses have similar BCR characteristics following each exposure to *P. falciparum*.

Between IgG^+^ B cells subsets, Morisita-Horn indexes were low (median: 0.2, range: 0.07–0.4, Fig. 8C), pointing to almost unique BCR repertoires in each of these populations. Over time, the Morisita-Horn index within populations did not increase (Fig. 8D), and no consistent changes in BCR repertoire characteristics were observed between IgG^+^ atypical and classical MBCs, most likely because only a small fraction of the total B cell subset is specific for *Plasmodium* antigens and will undergo activation and differentiation as a result of infection. While these results do not provide further insight into the origin of IgG^+^ atypical MBCs, it points to more extensive selection of IgG^+^ atypical MBCs by antigen or other stimuli as compared to IgM^+^ atypical MBCs and thus a more complex developmental pathway. Instead, the similarities in BCR characteristics between IgM^+^ atypical MBCs and naïve B cells are in line with the observations that IgM^+^ atypical MBCs may, at least in part, originate directly from the naïve B cell pool.

## Discussion

The atypical MBC population is expanded in individuals who have experienced *Plasmodium* infections [9, 10], but their relationship with other B cell subsets is unclear. In this study, a longitudinal analysis of the connectivity between B cell subsets in two children living in malaria-endemic Uganda was performed. BCR sequencing was used to track B cells during a malaria-free period and following an episode of clinical malaria, which allowed us to gain insight into the origin of atypical MBCs and their relationship with classical MBCs and naïve B cells.

In a previous study, it was observed that both classical and atypical MBCs contain FcRL5^−^ and FcRL5^+^ subsets [16]. Therefore, the effect of a malaria episode on FcRL5 expression in various B cell subsets was determined first. In agreement with other studies, FcRL5 expression was highest in IgG^+^ atypical MBCs [15, 16]. In contrast, among IgM^+^ B cells, activated MBCs had a higher frequency of FcRL5^+^ cells than other IgM^+^ subsets. Interestingly, FcRL5 expression consistently increased on all IgM^+^ memory B cells subsets after malaria, while results for IgG^+^ memory B cell subsets were less uniform. This difference may be related to the observation that the majority of *P. falciparum*-specific classical MBCs in children susceptible to malaria have not undergone class-switching [34]. Activation of parasite-specific IgM^+^ classical MBCs during infection might result in expansion of FcRL5^+^ IgM^+^ MBC subsets, in particular activated MBCs. Including activated MBCs in the BCR-seq experiment could have shed light on the relationship between activated MBCs and classical MBCs, but this was unfortunately not possible due to limited numbers of activated MBCs.

The observation that FcRL5 expression increased in all IgM^+^ MBC subsets after malaria raises the question of what the function of this marker is. FcRL5 has long been thought to be an inhibitory receptor. Work by Haga et al. [35] suggests that FcRL5 expression negatively impacts B cell activation by inhibiting signaling through the B cell receptor. Indeed, *Plasmodium*-associated atypical MBCs showed reduced BCR signaling upon BCR crosslinking in vitro as compared to naïve B cells and classical MBCs [15]. It has been proposed that the expression of inhibitory receptors, such as FcRL5, could be part of a feedback mechanism to control hypergammaglobulinemia or to downregulate the hyperactivated state of atypical MBCs [16, 36]. Recently, Ambegaonkar et al. [20] showed that activation of atypical MBCs expressing the inhibitory receptor FcγRIIB was restricted to membrane-bound antigen and did not occur in response to soluble antigen. In response to membrane-bound antigen, atypical MBCs actively excluded FcγRIIB from the immune synapse, resulting in robust BCR signaling and antigen internalization [20]. While the location of FcRL5 relative to the immune synapse was not determined, it is likely that, similar to FcγRIIB, FcRL5 is also excluded from the synapse, given its known negative effect on BCR signaling and plasma cell differentiation [15, 16]. As such, FcRL5 may contribute to the control of B cell responses during chronic antigen stimulation by preventing activation induced by soluble antigen and instead limit B cell activation to membrane-bound antigens.

Interestingly, FcRL5 was recently proposed to be a marker of durable B cell memory in response to tetanus and influenza immunization [37, 38]. Kim et al. [37] reported that classical MBCs and activated MBCs specific for the tetanus toxoid C fragment, a component of the tetanus vaccine, co-expressed FcRL5 and CD11c (another marker expressed by atypical MBCs), while the frequency of FcRL5^+^CD11c^+^ cells among bulk classical MBCs and activated MBCs was very low. These results suggest that markers previously associated with an atypical phenotype are expressed on long-lived antigen-specific memory B cells. In a non-peer-reviewed manuscript posted to BioRxiv, Nellore et al. [38] showed that FcRL5^+^CD11c^+^T-bet^+^ activated MBCs present shortly after primary influenza immunization give rise to plasmablasts upon secondary immunization a year later, providing further support for a role of FcRL5 and CD11c as markers of long-lived B cell memory. In this study, IgG^+^ atypical MBCs present after malaria seem to be predominantly derived from FcRL5^+^ IgG^+^ classical MBCs. Together with the observation that atypical MBCs readily respond to membrane-bound antigen [23], these results suggest that atypical MBCs may, in part, be seeded by long-lived memory B cells that are activated during *P. falciparum* infection and contribute to a functional antibody response against the parasite. This is seemingly in contrast with the observation that durable B cell memory takes a long time to develop in individuals living in endemic regions [10]. However, it is tempting to speculate that long-lived FcRL5^+^ classical MBCs undergo high rates of recall in response to frequent recurrent infections and are thus rapidly removed from the memory pool. This hypothesis is supported by a loss of clonal overlap between IgG^+^ classical MBCs over a 6-month period in this study (Fig. 3).

In contrast to IgG^+^ atypical MBCs, IgM^+^ atypical MBCs that were present after a malaria episode appeared to predominantly be derived from naïve B cells and FcRL5^−^ IgM^+^ classical MBCs. Relatedness of IgM^+^ atypical MBCs and naïve B cells is supported by recent findings that showed that the B cell receptor repertoires of IgM^+^ atypical MBCs and naïve B cells share many properties, including physicochemical properties of the HCDR3 and equal HCDR3 length, while substantial differences were observed for other B cell subsets [11]. In addition, this study showed that a large fraction of IgM^+^ atypical MBCs had not undergone somatic hypermutation. These findings suggest that the majority of IgM^+^ atypical MBCs have not gone through a germinal center reaction and may be derived from naïve B cells through extrafollicular activation following *P. falciparum* infection, similar to what has been suggested for activated naïve B cells in systemic lupus erythematosus patients [32]. The similarities in BCR repertoires between naïve B cells and IgM^+^ atypical MBCs suggest that this process can occur in the absence of antigen selection. Indeed, Aye et al. [13] recently showed that tetanus toxoid-specific B cells were recruited to the atypical MBC compartment as a result of *P. falciparum* exposure, indicative of bystander activation.

Strong connectivity between classical and atypical MBCs has previously been observed in malaria-experienced adults [15]. In this study by Portugal et al., classical and atypical MBCs showed no difference in clonal connections between IgM^+^ and IgG^+^ subsets, which may be related to the selection of adults with naturally acquired immunity against malaria in contrast to the semi-immune children included in this study or the timing of sample collection relative to a malaria episode. For example, the IgM response typically proceeds the IgG response, and IgM^+^ MBCs may therefore show increased expansion at a time point closer to *P. falciparum* infection. Alternatively, a later sampling time point could have resulted in lower expansion of and connections between IgG^+^ MBC subsets. More research will be needed to better understand potential differences between atypical MBCs induced in individuals of different ages and immune status and their dynamics over time.

Unfortunately, a limitation of this study is that B cell subsets were analysed in bulk without knowledge of antigen-specificity. While it was assumed that changes in clonal connections before and after a malaria episode were driven by *P. falciparum* infection, it is not possible to conclusively assign a functional role to atypical MBCs. This study setup may also explain why these results are different from those reported by Muellenbeck et al. [19], who did not observe overlap between *P. falciparum*-specific classical and atypical IgG^+^ MBCs. The small number of subjects included in this study and remaining questions surrounding the origin and functional role of atypical MBCs warrant further study of this topic.

## Conclusions

The results of this study point towards different dynamics and connectivity for IgM^+^ and IgG^+^ MBC cell subsets in response to a malaria episode. IgM^+^ atypical MBCs are short-lived cells that may be derived from naïve B cells and IgM^+^ FcRL5^−^ MBCs, while IgG^+^ atypical MBCs undergo higher rates of clonal expansion and appear most strongly connected with FcRL5^+^ IgG^+^ classical MBCs. Differences between IgM^+^ and IgG^+^ B cell subsets and between subsets with differential FcRL5 expression highlight the heterogeneity of the B cell compartment and the necessity to analyse these subsets independently.

## Supplementary Information

**Additional file 1:** **Table S1.** Antibodies used for the isolation of B cells by FACS. **Table S2.** Primers used for the generation of B cell receptor sequencing libraries. **Table S3.** Number of cells sorted for each B cell population. **Table S4.** Number of unique sequences in each sample. **Figure S1.** The calculation of clonal expansion and connection scores. **Figure S2.** Gating strategy for sorting naïve B cells, classical MBC, and atypical MBC populations. **Figure S3.** Percentages of CD19^hi^FcRL5^+^ B cells in all subsets at all time points for 3289. **Figure S4.** Percentages of CD19^hi^FcRL5^+^ B cells in all subsets at all time points for 3421. **Figure S5.** Changes in the percentage of CD19^hi^ FcRL5^+^ B cells over time.

## Data Availability

The data that supports the findings of this study are available for download at https://github.com/embunnik/clonal_scores.
