# Supplementary material for: Longitudinal analysis of FcRL5 expression and clonal relationships among classical and atypical memory B cells following malaria
Source: Malar J. 2021 Nov 10;20:435. doi: 10.1186/s12936-021-03970-1 (PMC8579674; doi:10.1186/s12936-021-03970-1)
Supplement: Supplementary file 1 — Additional file 1: Table S1. Antibodies used for the isolation of B cells by FACS. Table S2. Primers used for the generation of B cell receptor sequencing libraries. Table S3. Number of cells sorted for each B cell population. Table S4. Number of unique sequences in each sample. Figure S1. The calculation of clonal expansion and connection scores. Figure S2. Gating strategy for sorting naïve B cells, classical MBC, and atypical MBC populations. Figure S3. Percentages of CD19hiFcRL5+ B cells in all subsets at all time points for 3289. Figure S4. Percentages of CD19hiFcRL5+ B cells in all subsets at all time points for 3421. Figure S5. Changes in the percentage of CD19hi FcRL5+ B cells over time. [file 12936_2021_3970_MOESM1_ESM.pdf]

Supplementary Information to:

Longitudinal analysis of FcRL5 expression and clonal relationships among classical and atypical memory B cells following malaria

S. Jake Gonzales[1#], Sebastiaan Bol[1#], Ashley E. Braddom[1], Richard Sullivan[2,6], Raphael A. Reyes[1], Isaac Ssewanyana[3,4], Erica Eggers[5], Bryan Greenhouse[2], Evelien M. Bunnik[1]*

[1]Department of Microbiology, Immunology and Molecular Genetics, Long School of Medicine, The University of Texas Health Science Center at San Antonio, San Antonio, TX, USA

[2]Department of Medicine, University of California San Francisco, San Francisco, CA, USA

[3]London School of Hygiene and Tropical Medicine, London, UK

[4]Infectious Disease Research Collaboration, Kampala, Uganda

[5]UCSF Weill Institute for Neurosciences, Department of Neurology, University of California San Francisco, San Francisco, California, USA

[6]Present address: Shape Therapeutics, 219 Terry St., Seattle, WA, USA

[#] These authors contributed equally to this manuscript.

* Corresponding author: bunnik@uthscsa.edu

Table S1: Antibodies used for the isolation of B cells by FACS

Marker	Antibody clone	Vendor
CD3	UCHT1	Biolegend
CD14	M5E2	Biolegend
CD19	HIB19	Biolegend
CD20	B9E9	Beckman Coulter
CD21	Bu32	Biolegend
CD27	O323	Biolegend
IgM	MHM-88	Biolegend
IgG	G18-145	Biolegend
FcRL5	509f6	Biolegend

Table S2: Primers used for the generation of B cell receptor sequencing libraries

Name	Sequence
**Forward primers**	
General design	5'-trP1 adaptor-IGHV1-7-3'
VH1	5'CCTCTCTATGGGCAGTCGGTGAT-GAARRTYTCCTGCAAGGYWTC-3'
VH2	5'CCTCTCTATGGGCAGTCGGTGAT-CACRCTGACCTGCACCKTCTC-3'
VH3	5'CCTCTCTATGGGCAGTCGGTGAT-KARACTCTCCTGTRCAGCCTB-3'
VH4	5'CCTCTCTATGGGCAGTCGGTGAT-GTCCCTCACCTGCRCTGTCTM-3'
VH5	5'CCTCTCTATGGGCAGTCGGTGAT-GARGATCTCCTGTAAGGGTTC-3'
VH6	5'CCTCTCTATGGGCAGTCGGTGAT-CTCACTCACCTGTGCCATCTC-3'
VH7	5'CCTCTCTATGGGCAGTCGGTGAT-GAAGGTTTCCTGCAAGGCTTC-3'
**Reverse primers**	
General design	5'-adaptor-barcode+GAT key-IgG/M gene-specific sequence-3'
IgG	5'-CCATCTCATCCCTGCGTGTCTCCGACTCAG-barcode-GAT-GGGAAGACSGATGGGCCCTTGGTGG-3'
IgM	5'-CCATCTCATCCCTGCGTGTCTCCGACTCAG-barcode-GAT-GGAGTCGGGAAGGAAGTCCTGTGCGAG-3'

Table S3: Number of cells sorted for each B cell population.

B cell population	Time point (mo.)	3289	3421
Naïve B cells	0	91,291	22,906
	3	56,096	26,178
	6	63,457	46,539
FcRL5^{-} classical MBCs	0	8,434	7,099
	3	10,618	5,285
	6	14,284	7,578
FcRL5^{+} classical MBCs	0	4,017	581
	3	1,268	1,002
	6	5,985	1,726
FcRL5^{-} activated MBCs	0	708	1,118
	3	894	982
	6	410	1,178
FcRL5^{+} activated MBCs	0	354	385
	3	251	486
	6	2,070	559
FcRL5^{-} atypical MBCs	0	3,133	2,762
	3	1,614	2,822
	6	2,317	3,280
FcRL5^{+} atypical MBCs	0	850	2,547
	3	651	1,539
	6	4,198	2,732

MBCs, memory B cells; mo., months.

Table S4: Number of unique sequences in each sample.

		3289		3421	
B cell population	Time point (mo.)	IgM	IgG	IgM	IgG
Naïve B cells	0	5114	n.a.	5785	n.a.
	3	9453	n.a.	5217	n.a.
	6	3449	n.a.	7103	n.a.
FcRL5^{-} classical MBCs	0	820	120	342	81
	3	2251	350	1137	435
	6	1197	186	548	281
FcRL5^{+} classical MBCs	0	475	65	345	48
	3	2190	530	607	202
	6	2155	410	768	110
Atypical MBCs	0	1367	236	941	579
	3	708	492	581	236
	6	1486	711	1173	429

MBCs, memory B cells; mo., months.

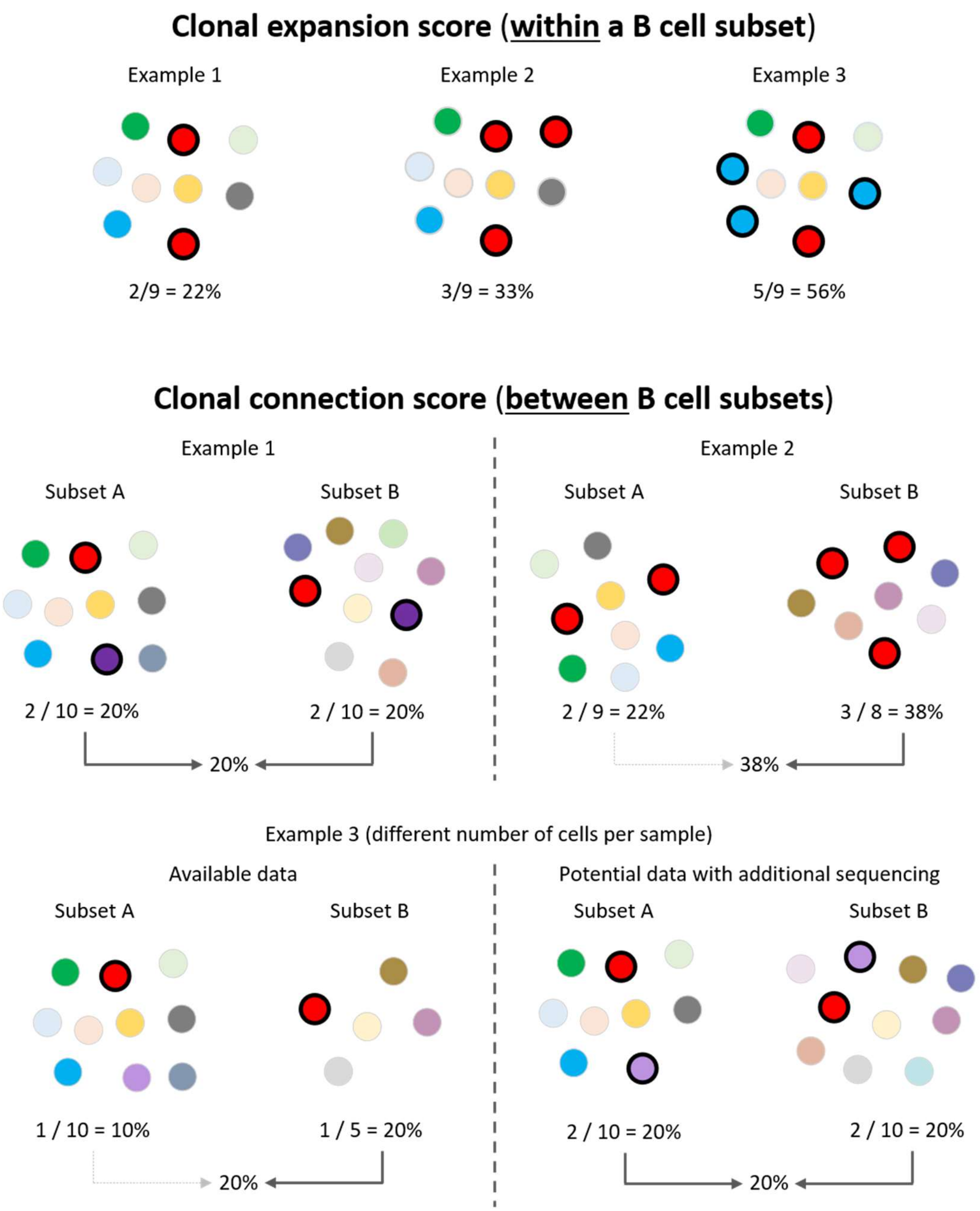

Figure S1: The calculation of clonal expansion and connection scores. Sequences are shown as circles. Sequences with dark black outline and same color form a clonotype. The top row shows examples of how clonal expansion scores (within B cell subsets) are calculated. The bottom part of the figures explains different scenarios of calculating clonal connection scores (between B cell subsets). Example 1 shows two subsets of an equal number of sequences and an equal number of sequences that are part of clonotypes. Examples 2 and 3 shows two subsets that differ in size and/or the number of clonotype sequences. In both cases (illustrated in more detail in example 3), the clonal connection score of the smaller subset (B) is used, because it is assumed that additional sequencing of subset B would have revealed additional sequences that match those in subset A (represented by the purple filled circles in the hypothetical situation on the right).

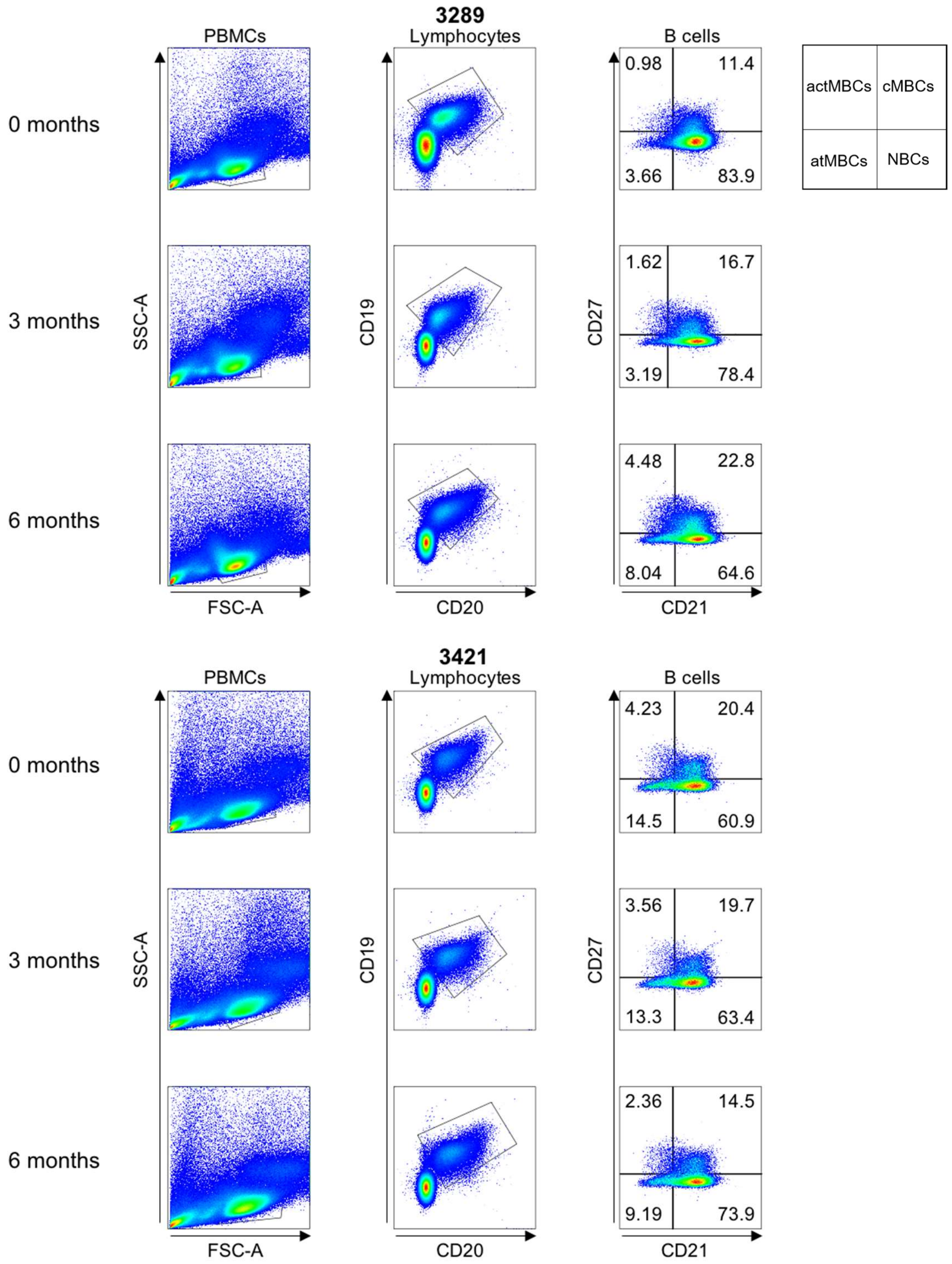

Figure S2: Gating strategy for sorting naïve B cells, classical MBC, and atypical MBC populations. PBMCs were gated on lymphocytes using the forward and sideward scatter. $CD19^{+}CD20^{+}$ B cells were then selected and divided into subpopulations based on CD21 and CD27 expression. NBCs, naïve B cells; cMBCs, classical memory B cells; actMBCs, activated memory B cells; atMBCs, atypical memory B cells.

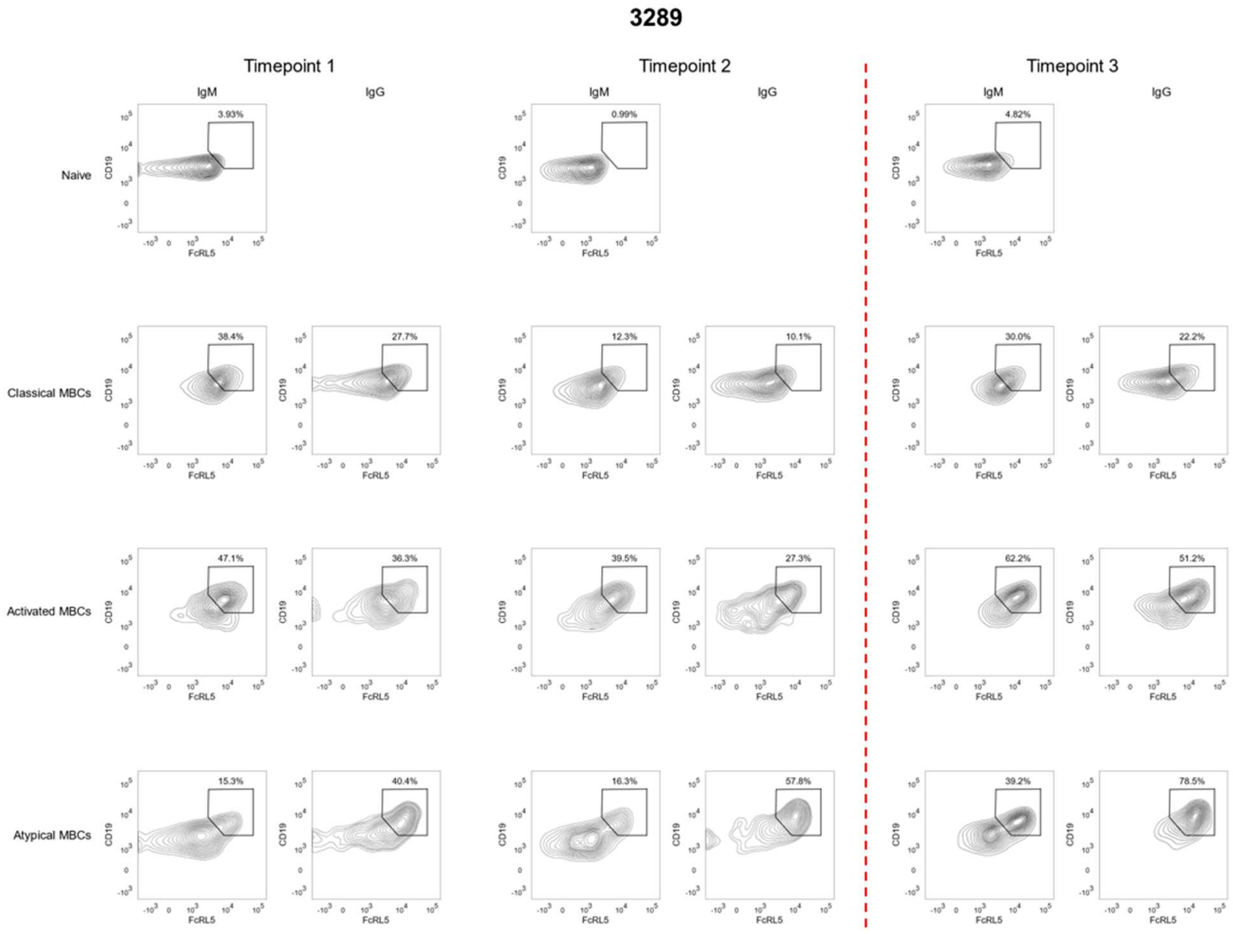

Figure S3: Percentages of CD19^hi^FcRL5^+^ B cells in all subsets at all time points for 3289. The red dashed line indicates the malaria episode between the second and third time point.

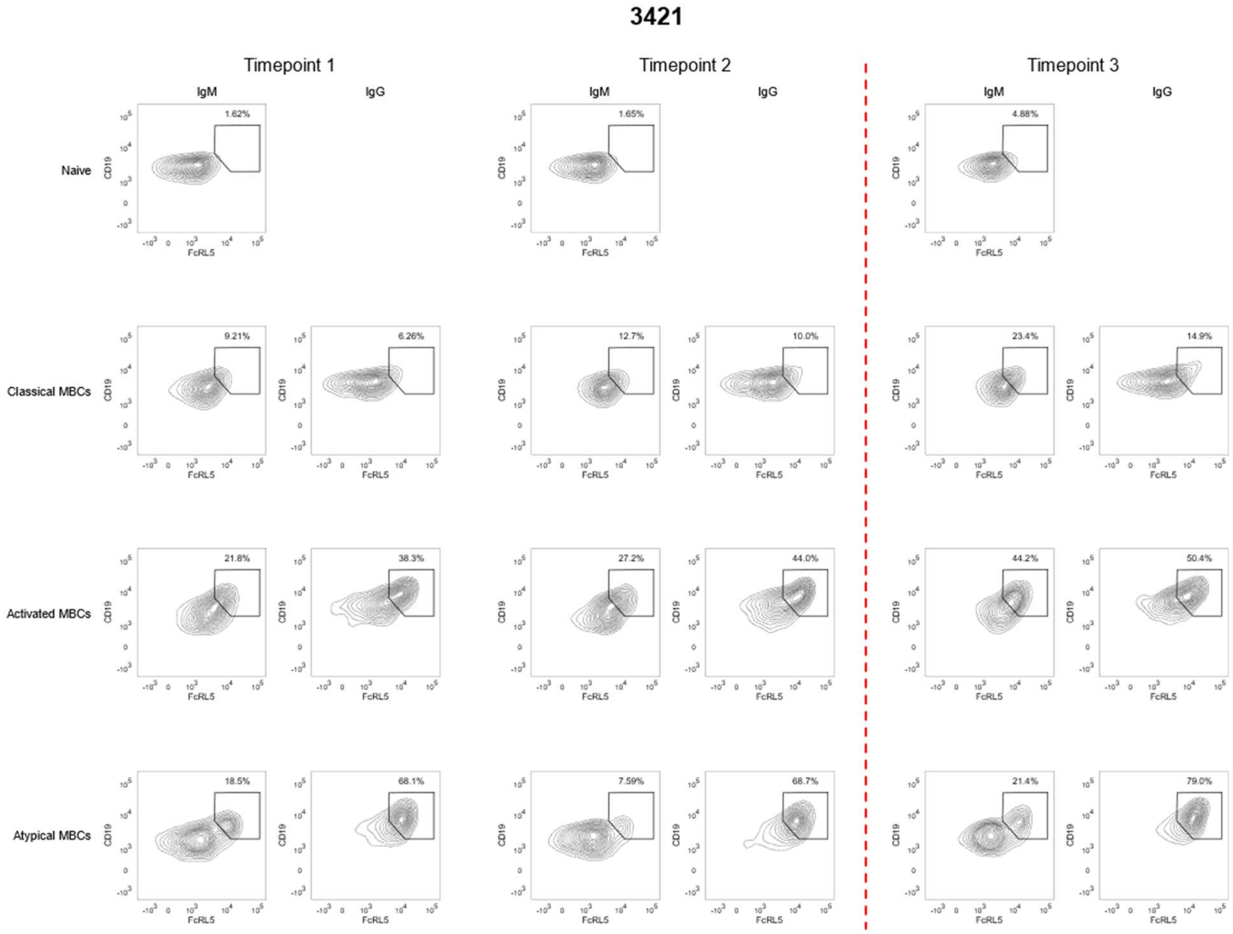

Figure S4: Percentages of CD19^{hi}FcRL5^{+} B cells in all subsets at all time points for 3421. The red dashed line indicates the malaria episode that occurred in this individual between the second and third time point.

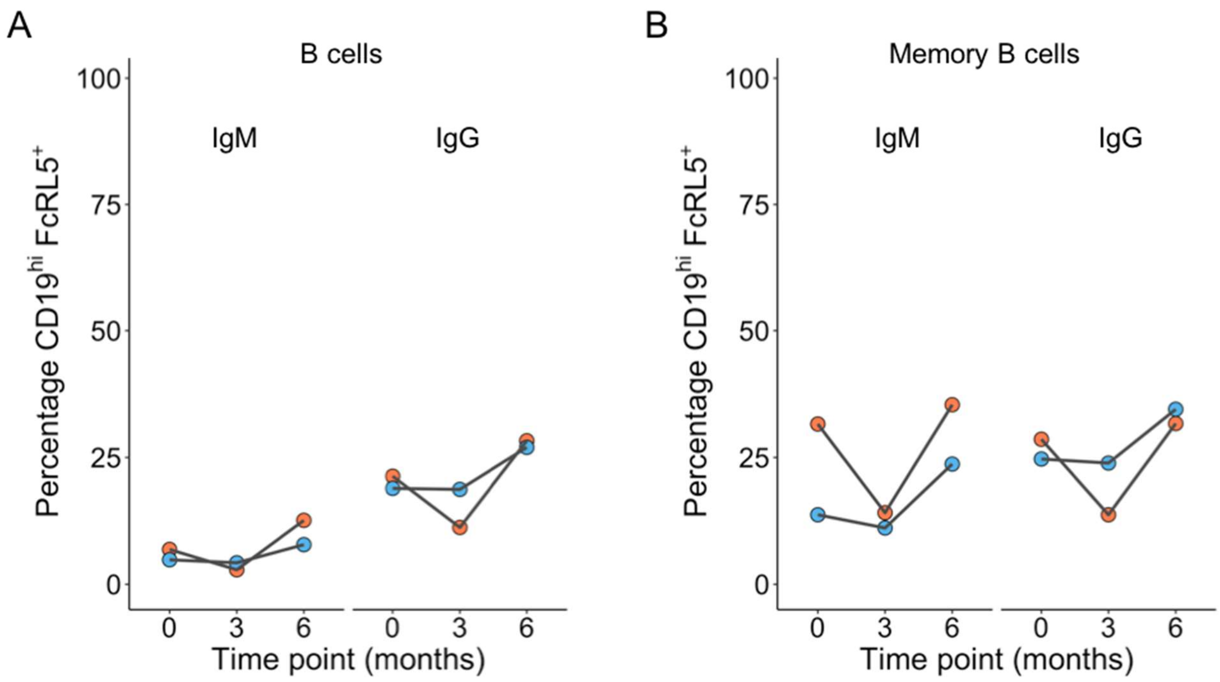

Figure S5: Changes in the percentage of CD19^{hi} FcRL5^{+} B cells over time. A) The percentage of IgM^{+} CD19^{hi} FcRL5^{+} B cells among all IgM^{+} B cells (left) and the percentage of all IgG^{+} CD19^{hi} FcRL5^{+} B cells among all IgG^{+} B cells (right). **B)** Same as in panel A, but showing the number of CD19^{hi} FcRL5^{+} B cells relative to memory B cells only. Data for child with cohort ID 3289 is shown in orange, while data for child with cohort ID 3421 is shown in blue.
